# Inadequate fine-tuning of protein synthesis and failure of amino acid homeostasis following inhibition of the ATPase VCP/p97

**DOI:** 10.1038/cddis.2015.373

**Published:** 2015-12-31

**Authors:** K Parzych, T M Chinn, Z Chen, S Loaiza, F Porsch, G N Valbuena, M F Kleijnen, A Karadimitris, E Gentleman, H C Keun, H W Auner

**Affiliations:** 1 Department of Medicine, Centre for Haematology, Imperial College London, London W12 0NN, UK; 2 Craniofacial Development and Stem Cell Biology, King's College London, London SE1 9RT, UK; 3 Faculty of Medicine, Department of Surgery and Cancer, Imperial College London, London W12 0NN, UK

## Abstract

The cellular mechanisms that control protein degradation may constitute a non-oncogenic cancer cell vulnerability and, therefore, a therapeutic target. Although this proposition is supported by the clinical success of proteasome inhibitors in some malignancies, most cancers are resistant to proteasome inhibition. The ATPase valosin-containing protein (VCP; p97) is an essential regulator of protein degradation in multiple pathways and has emerged as a target for cancer therapy. We found that pharmacological depletion of VCP enzymatic activity with mechanistically different inhibitors robustly induced proteotoxic stress in solid cancer and multiple myeloma cells, including cells that were insensitive, adapted, or clinically resistant to proteasome inhibition. VCP inhibition had an impact on two key regulators of protein synthesis, eukaryotic initiation factor 2*α* (eIF2*α*) and mechanistic target of rapamycin complex 1 (mTORC1), and attenuated global protein synthesis. However, a block on protein translation that was itself cytotoxic alleviated stress signaling and reduced cell death triggered by VCP inhibition. Some of the proteotoxic effects of VCP depletion depended on the eIF2*α* phosphatase, protein phosphatase 1 regulatory subunit 15A (PPP1R15A)/PP1c, but not on mTORC1, although there appeared to be cross-talk between them. Thus, cancer cell death following VCP inhibition was linked to inadequate fine-tuning of protein synthesis and activity of PPP1R15A/PP1c. VCP inhibitors also perturbed intracellular amino acid levels, activated eukaryotic translation initiation factor 2*α* kinase 4 (EIF2AK4), and enhanced cellular dependence on amino acid supplies, consistent with a failure of amino acid homeostasis. Many of the observed effects of VCP inhibition differed from the effects triggered by proteasome inhibition or by protein misfolding. Thus, depletion of VCP enzymatic activity triggers cancer cell death in part through inadequate regulation of protein synthesis and amino acid metabolism. The data provide novel insights into the maintenance of intracellular proteostasis by VCP and may have implications for the development of anti-cancer therapies.

The intracellular degradation of proteins that are damaged, misfolded, or no longer required is essential for normal cellular function. To maintain protein homeostasis (proteostasis), cells orchestrate a delicate balance between protein degradation and protein synthesis. Cancer cells may have a heightened dependence on protein degradation pathways, as their numerous genomic mutations often effect an imbalance in protein levels or the production of defective proteins.^1, 2^ Moreover, cancer cells may hyperactivate pathways that control protein synthesis, placing additional strain on the cellular mechanisms that govern protein degradation.^3, 4^ Therefore, drugs that disrupt protein breakdown pathways have considerable potential for anticancer therapy.

The ubiquitin–proteasome system (UPS) is the major mechanism in eukaryotic cells by which cytosolic, nuclear, and endoplasmic reticulum (ER)-derived proteins are degraded.^5^ Cells maintain physiological protein levels and an adequate intracellular amino acid pool by balancing protein synthesis with the activity of the UPS, and that of proteasome-independent degradation pathways.^6, 7^ The clinical use of proteasome inhibitors in multiple myeloma (MM) and mantle cell lymphoma has demonstrated that it is in principle possible to disrupt protein degradation in the UPS with fatal consequences for cancer cells, while largely sparing healthy cells. However, proteasome inhibitors are largely ineffective in other cancers.

VCP (valosin-containing protein; also known as p97) is an abundant ATPase that is conserved across all eukaryotes and is essential for life in budding yeast and mice.^8, 9, 10, 11^ VCP has the ability to use the energy derived from ATP hydrolysis to unfold client proteins, or to extract them from cellular structures. This allows VCP to engage in a range of cellular processes, but its role is best understood in the context of ER-associated degradation (ERAD).^12, 13, 14, 15, 16, 17, 18, 19^ As a key component of ERAD, VCP mediates the extraction of misfolded proteins across the ER membrane and their delivery to the proteasome.^20, 21, 22^ However, VCP has also been linked to the proteasome-independent handling of protein aggregates and autophagy.^23, 24, 25, 26, 27, 28^ Moreover, VCP has been implicated in proteasome recovery after proteasome inhibition, which may underlie the resistance of some cancers to proteasome inhibitors.^29, 30, 31^ Thus, VCP is fundamental for proteostasis. This broad involvement of VCP in intracellular protein turnover, combined with observations of aberrant VCP expression in different cancers,^32, 33, 34, 35, 36, 37, 38, 39, 40^ suggests that VCP inhibitors may overcome some limitations of proteasome inhibitors by affecting multiple proteostatic mechanisms simultaneously. Indeed, VCP-targeting compounds activate caspases and have an impact on both ubiquitin-dependent and autophagic pathways in cancer cells *in vitro* and *in vivo.*^27, 41, 42^ In contrast, primary rat hepatocytes and mouse skeletal muscle cells do not undergo apoptosis on VCP depletion and non-malignant human cells appear to be less susceptible to VCP inhibition than cancer cells.^27, 43, 44^ Phase I clinical trials of one VCP inhibitor are currently underway in patients with advanced solid cancers and relapsed/refractory myeloma (https://clinicaltrials.gov, NCT02243917 and NCT02223598). However, the mechanisms by which pharmacological VCP depletion induces cancer cell death, and how they differ from those mediated by proteasome inhibition, remain incompletely understood.

ER stress is potentially fatal to cells and can be brought about by various insults to the ER, such as the accumulation of misfolded proteins. It is linked to a diverse range of illnesses and is thought to be a key mechanism by which proteasome inhibitors effect their toxicity in MM cells.^45^ Cells normally respond to ER stress by activating the unfolded protein response (UPR), which comprises a number of cellular adaptations that aim to restore protein homeostasis.^46^ Phosphorylation of eukaryotic initiation factor 2*α* (eIF2*α*) on a single serine is central to one arm of the UPR and rebalances proteostasis by temporarily attenuating global messenger RNA (mRNA) translation.^47^ However, as a prolonged decrease in protein translation would prove fatal, phosphorylation of eIF2*α* also simultaneously triggers a negative feedback loop that promotes protein synthesis. This feedback loop begins with the preferential translation of the transcription factor activating transcription factor 4 (ATF4), which induces CCAAT/enhancer-binding proteins homologous protein (CHOP; encoded by the DNA damage-inducible transcript 3 (DDIT3) gene), another transcription factor. Both ATF4 and CHOP promote the expression of protein phosphatase 1 regulatory subunit 15A (PPP1R15A; also known as growth arrest and DNA damage-inducible protein, GADD34), the regulatory subunit of a stress-induced phosphatase that consists of PPP1R15A and PP1c. PPP1R15A/PP1c dephosphorylates eIF2*α* to reinstate physiological protein synthesis following the resolution of ER stress.^48, 49, 50, 51, 52, 53, 54^ However, in cells with unresolved ER stress this process generates oxidative stress and contributes to cell death.^51, 53, 54^ Thus, the eIF2*α*-ATF4/CHOP–PPP1R15A/PP1c feedback loop finely tunes protein synthesis to regulate cell viability under conditions of protein folding stress. eIF2*α* is also central to signaling networks that integrate oxidative stress and nutrient availability with other translation regulators such as mechanistic target of rapamycin complex 1 (mTORC1).^55, 56^

Here we studied the role of VCP in maintaining cancer cell proteostasis by using compounds that inhibit VCP enzymatic activity by different mechanisms.^27, 42^ We show that pharmacological depletion of VCP kills cancer cells of diverse tissue origins through mechanisms that regulate protein synthesis and amino acid homeostasis. Moreover, we demonstrate that the effects of VCP inhibition differ markedly from the effects of proteasome inhibition. The data provide previously unrecognized biological insights into the cellular mechanisms by which VCP governs proteostasis and may have important implications for the development of anticancer therapies.

## Results

### VCP inhibitors kill cancer cells independently of their tissue origins and sensitivity to proteasome inhibition

**Figure 1:**
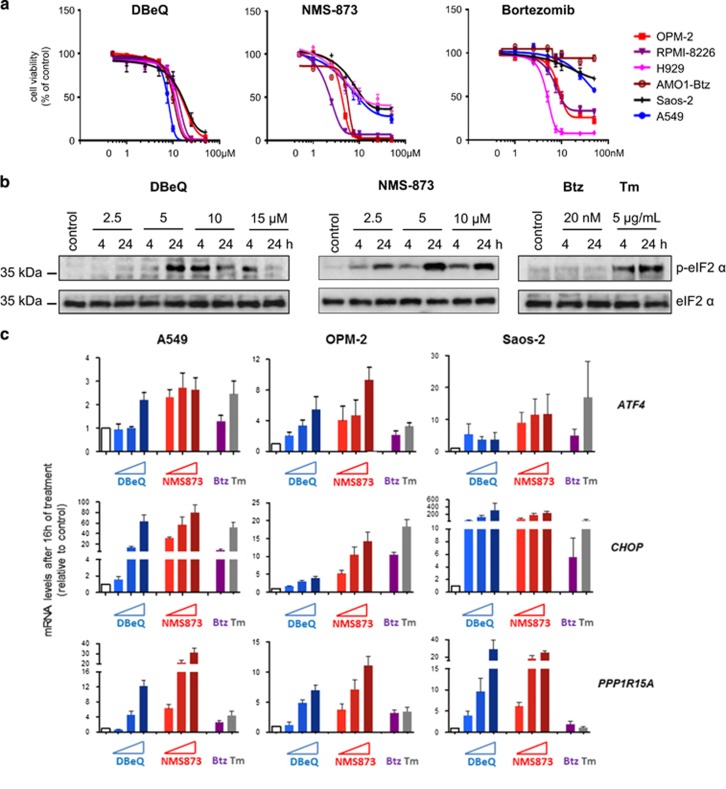
VCP inhibitors kill multiple myeloma and solid cancer cells and induce eIF2*α* signaling. **(a)** Viability of cancer cell lines after treatment with DBeQ, NMS-873, or bortezomib (Btz) for 24 h relative to untreated control cells. **(b)** Representative immunoblots with antibodies against phosphorylated (Serine 51) and total eIF2*α* on whole-cell extracts from A549 cells treated with DBeQ, NMS-873, bortezomib (Btz), or tunicamycin (Tm) as indicated. Treatment with 15 *μ*M NMS-873 for 24 h resulted in a protein yield that was too low for immunoblot analysis (not shown). **(c)** mRNA levels of the indicated eIF2*α*-regulated genes relative to control cells (white bars; solvent treated) determined by real-time quantitative PCR. The indicated lung cancer (A549), multiple myeloma (OPM-2), and osteosarcoma (Saos-2) cell lines were treated with DBeQ (5, 10, and 15 *μ*M), NMS-873 (5, 10, and 15 *μ*M), bortezomib (Btz, 20 nM), or tunicamycin (Tm, 5 *μ*g/ml) for 16 h. Data shown are the mean±S.E.M. from three independent experiments

We first examined the effects of VCP inhibition by directly comparing the ability of the VCP inhibitors *N*^2^*,N*^4^-dibenzylquinazoline-2,4-diamine (DBeQ)^27^ and Nerviano Medical Sciences-873 (NMS-873)^42^ with that of the proteasome inhibitor bortezomib, to kill a range of solid cancer and MM cell lines, as well as primary MM cells. We chose these two VCP-targeting compounds based on their extensive characterization and mechanistic difference. The quinazoline DBeQ is an ATP competitive VCP inhibitor, whereas NMS-873 is a non-ATP-competitive allosteric inhibitor of VCP.^1, 27, 42^ Both inhibitors rapidly turn off VCP enzymatic activity and thereby avoid potential limitations of genetic targeting related to slow or incomplete depletion of the abundant VCP.^1, 57^ As expected, a 24-h treatment with bortezomib effectively killed the OPM-2, Roswell Park Memorial Institute (RPMI)-8226, and H929 MM cell lines at an IC_50_ of ~5–10 nM (Figure 1a and Supplementary Table S1). However, bortezomib in concentrations up to 50 nM had a limited or no impact on the viability of A549 lung cancer cells, Saos-2 osteosarcoma cells, and bortezomib-adapted AMO1-Btz MM cells. In contrast, DBeQ and NMS-873 killed all cell lines tested within a narrow range of IC_50_ levels. The moderate differences in IC_50_s and in the level of cell death at 24 h that we observed can probably be explained by differences in the potency and efficacy of DBeQ and NMS-873 (Figure 1a and Supplementary Table S1).^1, 27, 42^ We also found that DBeQ- and NMS-873-induced cell death largely amounted to caspase-dependent apoptotic death (Supplementary Figure S1). Importantly, both inhibitors also killed primary bone marrow-derived MM cells from a patient with clinically bortezomib-resistant MM at concentrations similar to those that were effective in cell lines (Supplementary Table S1). Thus, an ATP-competitive and an allosteric VCP inhibitor effectively kill cancer cell lines of different tissue origins, including bortezomib-adapted and clinically bortezomib-resistant cells. The data suggest that the effects of VCP inhibitors have different mechanisms of action from proteasome inhibitors, and that their effects are not limited to cancer cells with a distinctive secretory load, such as MM cells.

### VCP inhibition activates eIF2*α* signaling and upregulates expression of its downstream targets

To investigate the role of eIF2*α*-ATF4/CHOP–PPP1R15A/PP1c signaling following VCP inhibition, we first examined the effects of DBeQ and NMS-873 on eIF2*α* phosphorylation. We found that both DBeQ and NMS-873 caused time- and dose-dependent phosphorylation of eIF2*α* (Figure 1b). Treatment with 15 *μ*M DBeQ also resulted in a decrease in eIF2*α* phosphorylation at 24 h compared with 4 h, possibly reflecting its dephosphorylation by PPP1R15A (Supplementary Figure S2). In contrast, bortezomib did not have a noticeable effect on eIF2*α* phosphorylation. However, bortezomib did cause intracellular accumulation of ubiquitinated proteins in A549 and OPM-2 cells, confirming that it was disrupting the UPS at the concentration used (Supplementary Figure S3). By comparison, tunicamycin, which induces ER stress by blocking *N*-linked glycosylation of ER-resident proteins, caused eIF2*α* phosphorylation after both 4 and 24 h (Figure 1b).

We next examined the effects of VCP and proteasome inhibition on key targets downstream of eIF2*α*. Using real-time quantitative PCR, we found that DBeQ and NMS-873 upregulated *CHOP*, *ATF4*, and *PPP1R15A* mRNA levels in a largely dose-dependent manner (Figure 1c). Consistent with its effect on cell death and eIF2*α* phosphorylation, NMS-873 appeared to be slightly more potent than DBeQ. We also found that the extent to which these genes were induced was largely comparable between lung cancer, osteosarcoma, and myeloma cells, although some differences between cell lines could be noted. In contrast to the VCP inhibitors, bortezomib had only a minimal effect on *PPP1R15A* expression in all three cell lines tested and a minor effect on *CHOP* expression in Saos-2 cells (Figure 1c). By comparison, tunicamycin upregulated *CHOP* and *ATF4* mRNA levels to a similar extent as the VCP inhibitors, but its effect on PPP1R15A expression was minimal. Both DBeQ and NMS-873 also increased CHOP and PPP1R15A protein levels, with NMS-873 again appearing more potent (Supplementary Figure S2). We then tested the ability of VCP inhibitors to induce eIF2*α* targets in bortezomib-adapted AMO1-Btz cells (Supplementary Figure S4). We found that NMS-873 and DBeQ strongly induced *CHOP* and *PPP1R15A* mRNA expression, and that NMS-873 also upregulated *ATF4*, similar to what we observed in A549, Saos-2, and OPM-2 cells. In contrast, bortezomib had no discernible effect and the effects of tunicamycin were limited.

When misfolded proteins accumulate in the ER, cells respond by creating more luminal chaperone proteins to assist in protein folding. This process is regulated by an alternative arm of the UPR from that involving eIF2*α* phosphorylation.^46^ To test whether VCP inhibitors also induced ER chaperones, we analyzed mRNA levels of binding immunoglobulin protein (*BIP*) and *P58IPK*, and found both to be largely upregulated in all three cell lines tested (Supplementary Figure S5). In line with our findings on eIF2*α* targets, NMS-873 had a greater effect on the transcriptional induction of *BIP* and *P58IPK* than DBeQ. Although tunicamycin also induced *BIP* and *P58IPK*, bortezomib had little effect. Finally, we tested whether VCP inhibitor-induced cell death correlated with the steady-state baseline expression of *VCP*, *ATF4*, *CHOP*, *PPP1R15A*, or *BIP* mRNAs in the cancer cell lines investigated and found no significant correlation (Supplementary Figure S6).

Taken together, these findings show pronounced effects of VCP inhibitors on the eIF2*α*-ATF4/CHOP-PPP1R15A/PP1c pathway and ER chaperones. Moreover, signaling downstream of eIF2*α* in response to VCP inhibitors appeared unaffected in bortezomib-adapted cells that show a blunted response to proteasome inhibition and ER stress. Our observations also demonstrate that the effects of DBeQ and NMS-873 on pathways employed by the UPR differ from those induced by bortezomib or tunicamycin, suggesting that VCP inhibition triggers a distinctive type of proteotoxic stress.

### Protein synthesis and PPP1R15A/PP1c-mediated eIF2*α* dephosphorylation modulate proteotoxic effects of VCP inhibition

**Figure 2:**
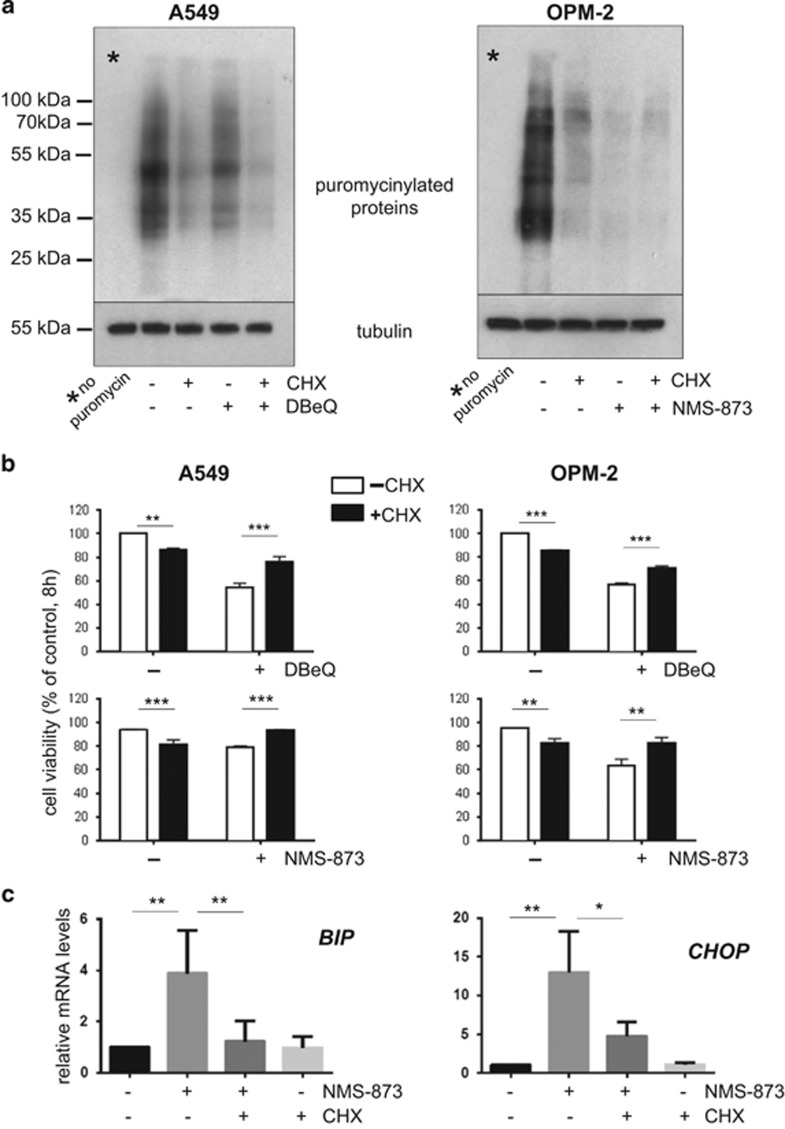
Protein synthesis modulates cell death and proteotoxic stress after VCP inhibition. **(a**) VCP inhibitors attenuate protein synthesis. Protein synthesis was monitored by immunoblotting for newly synthesized puromycinylated peptides. A549 and OPM-2 cells were treated for 8 h with DBeQ (15 *μ*M) or NMS (10 *μ*M) in the absence or presence of the translation inhibitor cycloheximide (CHX, 1 *μ*g/ml) before addition of puromycin for 10 min followed by the preparation of whole-cell extracts. **(b)** A block on protein synthesis reduces cell death following VCP inhibition. Cell viability of A549 and OPM-2 cells treated as described in (**a**). **(c)** Cycloheximide reduced VCP-inhibitor-mediated stress signaling. mRNA levels of the indicated genes following treatment of OPM-2 cells with NMS-873 (10 *μ*M) in the absence or presence of cycloheximide (CHX, 1 *μ*g/ml) for 8 h. Data shown in (**b**) and (**c**) are the mean±S.E.M. from three independent experiments. *P<0.05; **P<0.01; ***P<0.001

Protein synthesis can lead to cell death under conditions of ER stress.^54^ However, cells require controlled protein translation for normal function. This led us to investigate the role of protein synthesis following VCP inhibition, making use of the translation inhibitor cycloheximide. Immunoblotting for newly synthesized puromycinylated proteins demonstrated that a 8h treatment with cycloheximide largely abrogated protein synthesis, as expected. Our analysis also revealed that VCP inhibition triggered a considerable reduction in global protein translation (Figure 2a), a finding that is compatible with the phosphorylation of eIF2*α* we observed. We then determined that cycloheximide treatment resulted in cell death in a small proportion of cells (Figure 2b). However, translation inhibition with cycloheximide partially rescued DBeQ- and NMS-873-induced cell death. Moreover, cycloheximide rescue was associated with reduced induction of *CHOP* and *BIP* (Figure 2c). Thus, a block on protein synthesis that is itself cytotoxic has a cytoprotective effect on cells treated with VCP inhibitors that correlates with signs of reduced proteotoxic stress.

**Figure 3:**
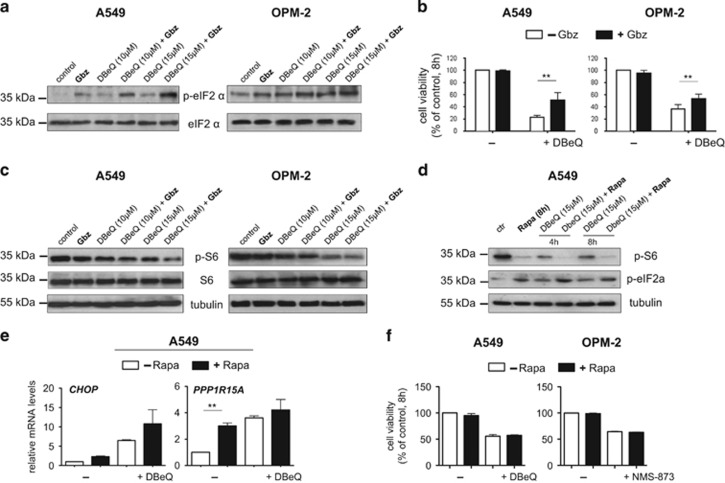
Impact of PPP1R15A/PP1c and mTORC1 signaling on proteotoxicity triggered by VCP inhibition. **(a)** Guanabenz treatment is associated with increased levels of eIF2*α* phosphorylation. Representative immunoblots with antibodies against phosphorylated (Serine 51) and total eIF2*α* on whole-cell extracts from A549 and OPM-2 cells treated for 8 h with DBeQ as indicated in the absence or presence of guanabenz (Gbz, 2.5 *μ*M). **(b)** Guanabenz reduces VCP inhibitor-mediated cell death. Cell viability after treatment with DBeQ (15 *μ*M) in the absence or presence of guanabenz (Gbz, 2.5 *μ*M) for 8 h. **(c)** Guanabenz decreases S6 phosphorylation as a readout for mTORC1 activity. Representative immunoblots with antibodies against phosphorylated (Serine 235/236) and total ribosomal protein S6 on whole-cell extracts from A549 cells after treatement with or without rapamycin (Rapa) as indicated. **(d)** mTORC1 inhibition has an impact on eIF2*α* phosphorylation. Immunoblottings demonstrating the effects of the mTORC1 inhibitor rapamycin (Rapa, 20 nM) on S6 and eIF2*α* phosphorylation in unstressed cells and cells treated with DBeQ. **(e)** The impact of mTORC1 inhibition on the indicated mRNA levels after VCP inhibition for 8 h. **(f**) Rapamycin does not modulate VCP inhibitor-induced cell death. Cell viability was determined after treatment with rapamycin (Rapa, 20 nM), DbeQ (15 *μ*M), and NMS-873 (10 *μ*M) for 8 h. Data shown in (**b**), (**e**), and (**f**) are the mean±S.E.M. from three independent experiments; **P<0.01

These data led us to examine whether the effects of VCP inhibition are modulated by the eIF2*α* phosphatase PPP1R15A/PP1c.^48, 49^ To this end we made use of the small molecule guanabenz, which selectively inhibits stress-induced dephosphorylation of eIF2*α* by PPP1R15A/PP1c.^58^ We first confirmed that treatment with guanabenz resulted in the expected increase in the level of eIF2*α* phosphorylation following VCP inhibition (Figure 3a). As expected, guanabenz largely increased expression of *CHOP*, *ATF4*, and *PPP1R15A* in DBeQ-treated cells but did not appear to affect expression levels of these genes in unstressed cells (Supplementary Figure S7). Next, we determined that DBeQ-induced cell death was indeed significantly attenuated in guanabenz-treated cells (Figure 3b), indicating that PPP1R15A/PP1c activity partly regulates cell death in response to VCP inhibition.

Considering its role as a regulator of protein synthesis, we then tested whether mTORC1 might be affected by PPP1R15A/PP1c. We found that DBeQ alone caused a moderate reduction in ribosomal protein S6 phosphorylation, a readout for mTORC1 activity (Figure 3c).^6, 59^ We also observed that co-treatment with guanabenz mildly enhanced the DBeQ-induced reduction of S6 phosphorylation. Thus, inhibition of PPP1R15A/PP1c-mediated eIF2*α* dephosphorylation by guanabenz decreases cell death and enhances attenuation of mTORC1 signaling following VCP inhibition.

We then tested whether inhibition of mTORC1 activity with rapamycin had an impact on eIF2*α* phosphorylation and the proteotoxic effects of VCP depletion. Indicating its inhibitory effect on mTORC1, rapamycin remarkably reduced phosphorylation of S6 in unstressed A459 cells and appeared to mildly enhance the reduction of S6 phosphorylation in cells treated with DBeQ (Figure 3d). Rapamycin also increased eIF2*α* phosphorylation both in unstressed and DBeQ-treated cells, although the effect appeared moderate (Figure 3d). Moreover, rapamycin appeared to increase mRNA levels of *CHOP* and *PPP1R15A*, in line with its effects on eIF2*α* phosphorylation (Figure 3e). Comparable results on the effects of rapamycin on eIF2*α* phosphorylation and gene expression were observed in OPM-2 cells treated with NMS-873 (not shown). Importantly, rapamycin had no effect on the viability of cells treated with either DBeQ or NMS-873 (Figure 3f). Thus, VCP inhibition has an impact on two important cellular regulators of protein synthesis, eIF2*α* and mTORC1, and there appears to be cross-talk between them; however, mTORC1 does not affect cell viability following VCP inhibition.

### VCP inhibition disrupts intracellular amino acid homeostasis

**Figure 4:**
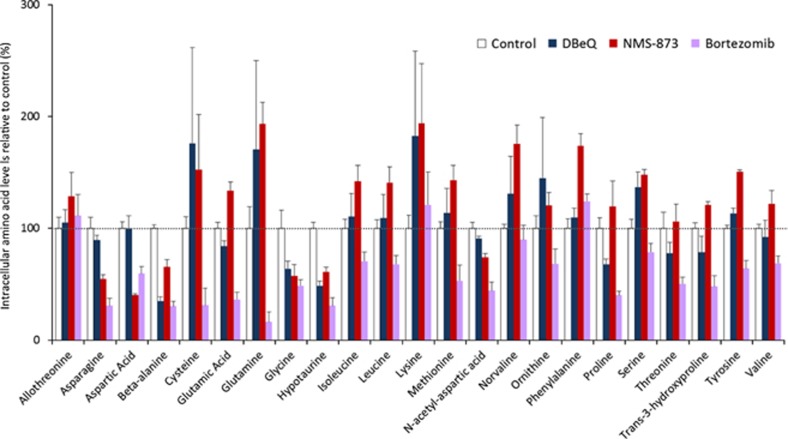
Inhibitors of VCP and the proteasome have different effects on intracellular amino acid levels. Relative levels of intracellular amino acids determined by GC-MS. A549 cell extracts were prepared as described in Materials and Methods after treatment with DBeQ (10 *μ*M), NMS-873 (10 *μ*M), or bortezomib (20 nM) for 8 h. Data shown are the mean±S.E.M. relative to untreated (control) cells from four independent experiments

To maintain intracellular amino acid homeostasis, cells tightly co-ordinate protein synthesis with degradation.^6^ Proteasome inhibition has been reported to reduce intracellular amino acids levels;^60, 61^ however, the effects of VCP inhibitors on amino acid homeostasis have not been studied. To address this gap in knowledge, we performed unsupervised gas chromatography–mass spectrometry (GC-MS) of extracts from A549 cells treated with DBeQ or NMS-873 and compared their effects with that of bortezomib (Figure 4 and Supplementary Table S2). Bortezomib decreased intracellular levels of numerous amino acids, consistent with previous reports.^6, 60, 61^ However, VCP inhibitors had a different and more complex effect. Although both DBeQ and NMS-873 caused a significant decrease of *β*-alanine and hypotaurine levels, treatment with NMS-873 also decreased levels of asparagine, aspartic acid, and *N*-acetyl aspartic acid. Surprisingly, treatment with NMS-873 significantly increased the levels of four other amino acids: glutamic acid, phenylalanine, serine, and tyrosine. DBeQ appeared to increase the levels of three of these four amino acids, and those of several others, but the differences were not statistically significant compared with untreated cells (Supplementary Table S2). Although DBeQ and NMS-873 caused changes in intracellular amino acid levels that were largely comparable with each other, a larger number of statistically significant changes were observed after NMS-873 treatment, which may be explained by its higher potency. Thus, pharmacological depletion of VCP enzymatic activity elicits a complex effect on intracellular amino acid pools.

**Figure 5:**
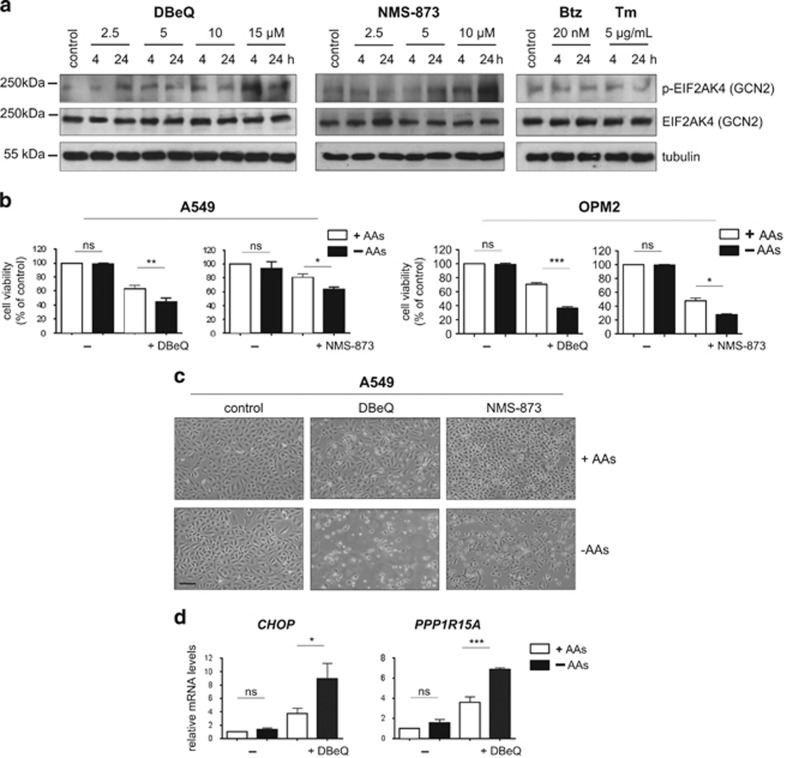
VCP inhibitors disrupt intracellular amino acid homeostasis. **(a)** VCP inhibitors trigger EIF2AK4 phosphorylation. Representative immunoblots with antibodies against phosphorylated (Thr889) and total EIF2AK4 (GCN2) on whole-cell extracts from A549 cells treated with DBeQ, NMS-873, bortezomib (Btz), or tunicamycin (Tm). **(b)** Cell viability of A549 and OPM-2 cells growing in complete media (+AA) or media deficient in L-glutamine, L-methionine, and L-cystine (–AA). Cells were treated with DBeQ (15 *μ*M for A549 cells and 10 *μ*M for OPM2 cells) or NMS-873 (10 *μ*M for A549 cells and 5 *μ*M for OPM-2 cells) for 16 h. **(c)** Representative images of A549 cells treated as in (**b**). Images shown were taken with an ECOS core digital microscope at × 100 magnification (size bar: 25 *μ*m). **(d)** mRNA levels of the indicated genes relative to untreated controls determined by real-time quantitative PCR after treatment of A549 cells as described in (**b**). Data shown in (**b**) and (**d**) are the mean±S.E.M. from three independent experiments. *P<0.05, **P<0.01, ***P<0.001

We next asked whether the effect of VCP inhibition on amino acid levels was functionally relevant to cells. To test this, we first examined activation of eukaryotic translation initiation factor 2*α* kinase (EIF2AK4; general control nonderepressible 2 (GCN2)), an eIF2*α* kinase primarily activated by amino acid deprivation.^62, 63, 64^ Indeed, immunoblots on extracts from A549 cells treated with DBeQ and NMS-873 showed phosphorylation of EIF2AK4 at Threonine 889, indicating its activation. In contrast, neither bortezomib nor tunicamycin appeared to trigger phosphorylation of EIF2AK4 (Figure 5a).

We then hypothesized that putting an additional strain on amino acid metabolism would enhance the toxicity of VCP inhibitors if their effects on amino acid levels were functionally relevant. Indeed, when we depleted three amino acids (L-methionine, L-cystine, and L-glutamine) from the cell culture medium, we observed increased cell death on treatment with DBeQ and NMS-873 (Figures 5b and c). Moreover, we observed that amino acid depletion enhanced the VCP inhibitor-mediated induction of *CHOP* and *PPP1R15A* (Figure 5d). However, amino acid depletion affected neither viability nor *CHOP*/*PPP1R15A* expression in unstressed cells or in cells treated with tunicamycin (Figure 5d and Supplementary Figure S8). Thus, VCP inhibitors disrupt intracellular amino acid homeostasis and enhance cellular dependence on amino acid supplies.

## Discussion

Phosphorylation of the translation initiation factor eIF2*α* can promote cell survival under acute stress conditions by attenuating protein translation. However, eIF2*α* phosphorylation can also mediate death in cells with unresolved stress by promoting protein synthesis.^46, 54^ We found that VCP inhibitors consistently and strongly activated eIF2*α* signaling in cancer cells derived from different tissues, including proteasome inhibitor-adapted myeloma cells. This finding led us to investigate the role of major protein translation control pathways in VCP inhibitor-induced cell death. We found that a block on protein translation, which was toxic on its own, was cytoprotective in cells in which VCP was inhibited. How could protein synthesis have a detrimental impact on cells? The effect might be explained by a reduced cellular dependence on protein quality control mechanisms that depend on VCP, such as ERAD, when protein synthesis is reduced. Protein synthesis under conditions of VCP inhibition could also result in higher level of reactive oxygen species or loss of ATP.^54^ Intriguingly, protein synthesis was attenuated in response to VCP inhibition, probably as part of the proteotoxic stress response. Therefore, our data suggest that either the extent or nature of this downregulation was inadequate for optimal cytoprotection. This finding highlights the requirement for cells to precisely fine-tune protein synthesis under conditions of proteotoxic stress.

Our observations also indicate that VCP and PPP1R15A/PP1c have an impact on the mTORC1 pathway, although it remains to be established how these effects are mediated. Moreover, we found that pharmacological inhibition of mTORC1 had an impact on eIF2*α* and its downstream targets. These data are important, as they show that at least two key cellular signaling hubs that regulate protein synthesis, eIF2*α* and mTORC1, are affected by VCP inhibition, and that there is cross-talk between them, thereby adding to the growing literature on the interactions between eIF2*α* and mTORC1.^55, 64, 65, 66, 67^ However, only guanabenz, but not rapamycin, had a cytoprotective effect following VCP inhibition. This may be explained by feedback effects of mTORC1 inhibition on the PI3K/Akt pathway and mTORC2, or effects on autophagy, which is also modulated by VCP.^27, 42, 55, 64^ The findings may be clinically relevant, as some approaches to improve cancer therapy aim to reduce the activity of pathways that promote protein synthesis, such as mTORC1.^68^ Our results suggest that such a strategy may not be beneficial when combined with proteotoxic treatment approaches such as VCP inhibition.

The effects of VCP inhibitors on mTORC1, which also responds to amino acid shortage, led us to investigate their effects on intracellular amino acid homeostasis. We found that VCP inhibitors triggered both decreases and increases in individual intracellular amino acid levels. In contrast, and in line with previous reports, we found that proteasome inhibition resulted in a substantial decrease in the intracellular levels of many amino acids^6, 60^ The unexpected impact of VCP inhibition on amino acid levels might be related to the VCP inhibitors' complex effects on the regulation of protein synthesis and thus amino acid utilization. Given that DBeQ and NMS-873 promoted phosphorylation of EIF2AK4, a major eIF2*α* kinase that senses amino acid shortages,^62^ the complex effects on amino acid homeostasis elicited by VCP inhibitors are likely to be functionally relevant. In contrast, bortezomib did not induce EIF2AK4 phosphorylation and was less effective than the VCP inhibitors in inducing cell death, suggesting that the cells activated mechanisms that at least partly compensated for the reduction in amino acid levels triggered by proteasome inhibition.

We noted that EIF2AK4 phosphorylation occurred quite some time after the changes in mTORC1 activity and eIF2*α* phosphorylation. As such, the precise mechanisms and kinetics by which VCP inhibition leads to EIF2AK4 activation remain to be established. Moreover, it is still unclear whether EIF2AK4 or the ER stress sensor EIF2AK3 protein kinase R (PKR)-like endoplasmic reticulum kinase is the predominant eIF2*α* kinase after VCP inhibition or, indeed, whether both have a role.

We also found that the toxic effects of VCP inhibition were enhanced when extrinsic amino acid supplies were limited, whereas the same amino acid limitation had no measurable effect on unstressed cell or cells undergoing protein misfolding stress. Given that tumours may be hyperdependent on nutrient supplies and may scavenge extracellular protein to ensure sufficient amino acid availability,^69^ the VCP inhibitor-associated heightened dependence on amino acid supplies may be clinically relevant. Taken together, our data provide evidence that VCP inhibitors induce a functionally detrimental disruption of intracellular amino acid homeostasis.

**Figure 6:**
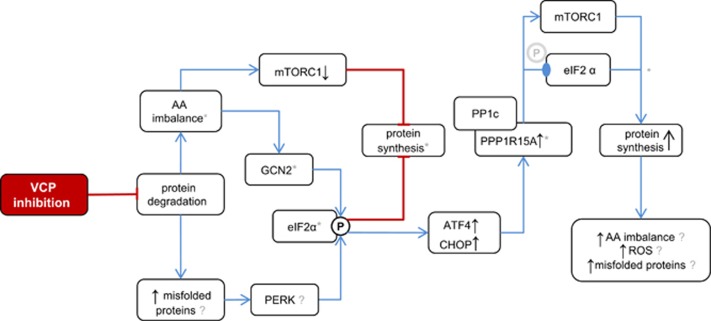
Proposed working model of the mechanisms by which VCP inhibitors induce cancer cell death. VCP inhibition impinges on protein degradation, resulting in aberrant amino acid (AA) recycling and increased levels of misfolded proteins in the ER. This results in reduced mTORC1 activity and activates the eIF2*α* kinases EIF2AK4 (GCN2) and EIF2AK3 (PERK), thereby attenuating protein synthesis to alleviate proteotoxic stress. Downstream of eIF2*α*, the ATF4/CHOP-mediated induction of PPP1R15A governs eIF2*α* dephosphorylation by PPP1R15A/PP1c and increases mTORC1 activity. Together, these effects promote protein synthesis, resulting in inadequate attenuation of protein translation that aggravates cell stress and death through mechanisms that may involve amino acid imbalance, reactive oxygen species, and ER stress caused by misfolded proteins. 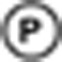
Phosphorylated serine 51; 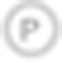
dephosphorylated serine 51; *probable qualitative or quantitative differences between VCP and proteasome inhibitors

In summary (Figure 6), we demonstrate that compounds that target VCP enzymatic activity disrupt multiple aspects of intracellular protein metabolism. These findings advance our understanding of how VCP maintains proteostasis and may be relevant for the clinical development of VCP inhibitors for cancer therapy.

## Materials and Methods

### Cells and reagents

The human MM cell lines OPM-2, H929, and RPMI-8226 were purchased from the Deutsche Sammlung von Mikroorganismen und Zellkulturen GmbH (Braunschweig, Germany). Saos-2 cells were from the European Collection of Cell Cultures (Salisbury, UK). A549 cells were a gift from Jane Mitchell (Imperial College London, London, UK). Cell culture identity was verified by short tandem repeat profiling provided by the University of Sheffield, UK (not shown). Bortezomib-adapted AMO-1 cells were a gift from Christoph Driessen (Kantonsspital St. Gallen) and have been described before.^70, 71^ Cell lines were regularly tested for mycoplasma contamination using the MycoAlert Mycoplasma Detection Kit (Lonza, Cambridge, UK). Primary human myeloma cells were isolated from a diagnostic bone marrow aspirate performed on a patient with relapsed MM by CD138 magnetic bead selection under appropriate research ethics committee approval (REC reference 11/H0308/9) at Imperial College Healthcare NHS Trust. The cells were isolated shortly before initiation of treatment with a regimen consisting of bortezomib, cyclophosphamide, and dexamethasone, to which the patient subsequently proved to be clinically resistant. Cells were grown in RPMI (myeloma cells) or Dulbecco's modified Eagle's medium (DMEM; Saos-2, A549 cells) media (Sigma-Aldrich, St. Louis, MO, USA) supplemented with 10% fetal bovine serum (FBS) and penicillin plus streptomycin. For experiments on amino acid depletion we compared DMEM and RPMI containing amino acids (Sigma-Aldrich; D1145, R8758) with DMEM and RPMI without L-methionine, L-cystine, and L-glutamine (Sigma-Aldrich; D0422, R7513), both supplemented with 10% dialyzed FBS (Sigma-Aldrich; F0392). DBeQ (Biovision, Milpitas, CA, USA), NMS-873 (Sigma-Aldrich), bortezomib (Sigma-Aldrich), tunicamycin (Sigma-Aldrich), guanabenz (Sigma-Aldrich), rapamycin (Sigma-Aldrich), cycloheximide (Sigma-Aldrich) and Z-VAD-FMK (Tocris Bioscience, Bristol, UK) were dissolved in dimethylsulfoxide and stored at −20 °C.

### Cell viability

Cell viability was measured using the AlamarBlue cell viability reagent (Thermo Fisher, Waltham, MA, USA) according to the manufacturer's instructions. Briefly, cells were grown in 96-well plates, AlamarBlue was added at 1/10 of the culture volume at the end of the indicated experimental treatment times, and the fluorescence read at an emission wavelength of 590 nm following excitation at 544 nm. Apoptosis was determined by flow cytometric analysis of Annexin V-FITC and propidium iodide-stained cells (BD Biosciences, San Jose, CA, USA). All results were obtained from three independent experiments, each consisting of three technical replicates. Results are shown as mean viability relative to untreated or vehicle (solvent)-treated cells.

### mRNA expression analysis by real-time PCR

Cells were collected and snap frozen using liquid nitrogen. RNA was extracted using the GeneJET RNA Purification Kit (Thermo Fisher) followed by removal of genomic DNA according to the manufacturer's instructions. cDNA synthesis was performed using the RevertAid First Strand cDNA Synthesis Kit (Thermo Fisher) according to the manufacturer's instructions using an Applied Biosystems 2720 Thermal Cycler (Life Technologies, Carlsbad, CA, USA). PCR reactions were performed on an Applied Biosystems StepOnePlus machine (Applied Biosystems, Foster City, CA, USA) using 10 *μ*l SYBR Green JumpStart Taq ready Mix (Sigma-Aldrich), 0.3 *μ*M sequence-specific primers, and 25 ng cDNA under standard conditions.

### Immunoblotting

Whole-cell protein extracts were prepared on ice using a lysis buffer containing 50 mM HEPES (N-2-hydroxyethylpiperazine-*N′*-2-ethanesulfonic acid) pH 7.5, 50 mM NaF, 5 mM Na pyrophosphate, 1 mM EDTA, 1 mM dithiothreitol, 10% glycerol, 1% Triton, and Complete EDTA-free Protease Inhibitor Cocktail (Roche, Basel, Switzerland). Primary antibodies used were as follows: phospho-GCN2 Thr889 (Abcam, Cambridge, UK), GCN2 (Cell Signalling Technology, Danvers, MA, USA), GADD34/PPP1R15A (Santa Cruz Biotechnology, Dallas, TX, USA), phospho-eIF2*α* Ser51 (Cell Signalling Technology), eIF2*α* (Cell Signalling Technology), *β*-tubulin (Cell Signalling Technology), S6 ribosomal protein 5G10 (Cell Signalling Technology), and phospho-S6 ribosomal protein Ser235/236 (Cell Signalling Technology). The PageRuler Plus Prestained Protein Ladder (Thermo Fisher) was used as a molecular weight marker.

### GC-MS of intracellular amino acids

Intracellular metabolites were extracted from cultured cells by cold methanol quenching. Aqueous metabolites were separated from the intracellular extract using a 2 : 1 : 3 chloroform:methanol:water extraction method. The aqueous portion of the extract was separated and lyophilized in silanized 1.5 ml glass vials before analysis. Derivatization for GC-MS was carried out by methoximation followed by trimethylsilylation according to the protocol described by Kind *et al.*^72^ Samples were analyzed on an Agilent 7890 gas chromatograph (Agilent Technologies, Santa Clara, CA, USA) connected to an Agilent 5975 MSD (Agilent Technologies) using the FiehnLib settings^72^ and retention time-locking to myristic acid-d27. GC-MS data were processed by deconvolution using AMDIS using the Fiehn library, followed by integration using GaVIN^73^ based on the quantification ion for each metabolite as taken from the Fiehn library. Data were normalized to cell number and statistical analyses were carried out in the R statistical environment.

### Analysis of protein synthesis by puromycin labeling

Semi-quantitative monitoring of protein synthesis was carried out based on the previously described SUnSET method.^74^ Briefly, newly synthesized peptides were labeled in cultured cells by the addition of puromycin (InvivoGen, San Diego, CA, USA); 5 *μ*g/ml for 10 min before cells were collected and whole-cell extracts were prepared for immunoblotting as described above, using anti-puromycin antibody clone 12D10 (Merck Millipore, Darmstadt, Germany) and anti-mouse IgG-HRP-linked antibody (Cell Signalling Technology).

### Statistical analysis

Unless stated otherwise, data show means and S.E.M. from three independent experiments. Analysis of variance and Bonferroni posttests were used for comparisons between treatment groups. Correlation between cell viability loss after inhibitor treatment and baseline gene expression was analyzed using Pearson's correlation coefficient (*r*). *T*-test was used to establish correlation coefficient significance. Calculations were done using GraphPad PRISM Version 6.05 (GraphPad Software, La Jolla, CA, USA).

## Abbreviations

ATF4: activating transcription factor 4
BIP: binding immunoglobulin protein
CHOP: CCAAT/enhancer-binding protein homologous protein
DBeQ: *N*^*2*^*,N*^*4*^-dibenzylquinazoline-2,4-diamine
DMEM: Dulbecco's modified Eagle's medium
eIF2*α*: eukaryotic initiation factor 2*α*
EIF2AK: eukaryotic translation initiation factor 2*α* kinase
ER: endoplasmic reticulum
ERAD: ER-associated degradation
FBS: fetal bovine serum
GADD34: growth arrest and DNA damage protein
GC-MS: gas chromatography–mass spectrometry
GCN2: general control nonderepressible 2
HEPES: *N*-2-hydroxyethylpiperazine-*N*′-2-ethanesulfonic acid
MM: multiple myeloma
mRNA: messenger RNA
mTORC: mechanistic target of rapamycin complex
NMS-873: Nerviano Medical Sciences-873
PPP1R15A: protein phosphatase 1 regulatory subunit 15A
RPMI: Roswell Park Memorial Institute
UPR: unfolded protein response
UPS: ubiquitin–proteasome system
VCP: valosin-containing protein
