# Supplementary material for: Inadequate fine-tuning of protein synthesis and failure of amino acid homeostasis following inhibition of the ATPase VCP/p97
Source: Cell Death Dis. 2015 Dec 31;6(12):e2031–. doi: 10.1038/cddis.2015.373 (PMC4720905; doi:10.1038/cddis.2015.373)
Supplement: Supplementary Figures [file cddis2015373x1.pdf]

Supplementary Figure S1.

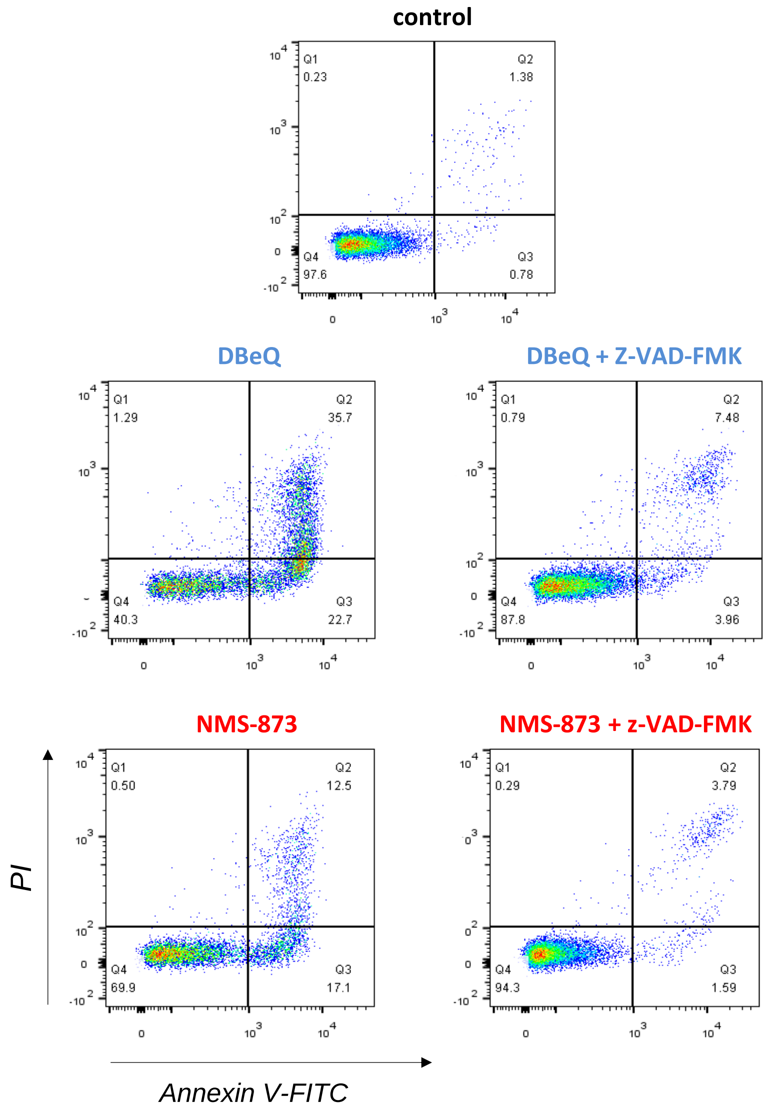

Supplementary Figure S1. Cell death induced by VCP inhibition is largely caspase-dependent. Cell death of A549 cells was determined by FACS analysis of cells stained with Annexin V-FITC and propidium iodide (PI) after treatment with DBeQ (17.5µM) or NMS-873 (15µM), with or without the pan-caspase inhibitor z-VAD-FMK (50µM), for 24h.

Supplementary Figure S2.

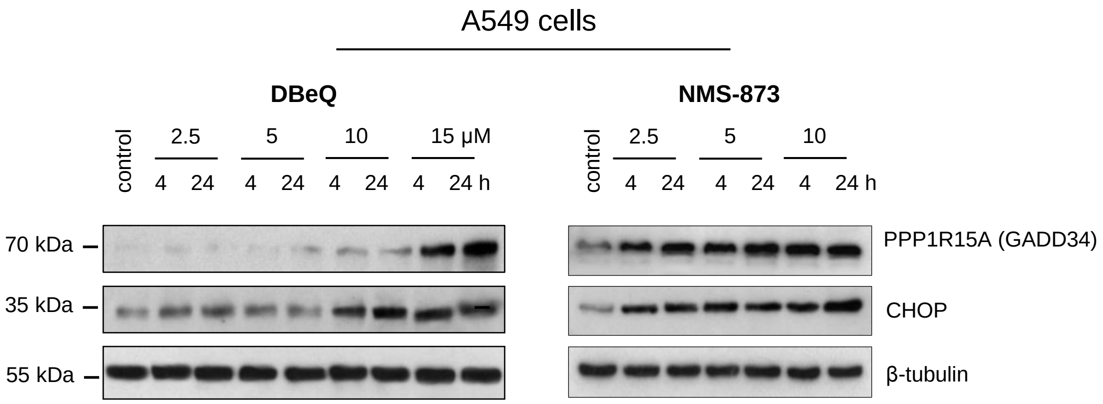

Supplementary Figure S2. VCP inhibition induces PPP1R15A (GADD34) and CHOP protein levels. Representative immunoblots on whole cell extracts from A549 cells are shown.

Supplementary Figure S3.

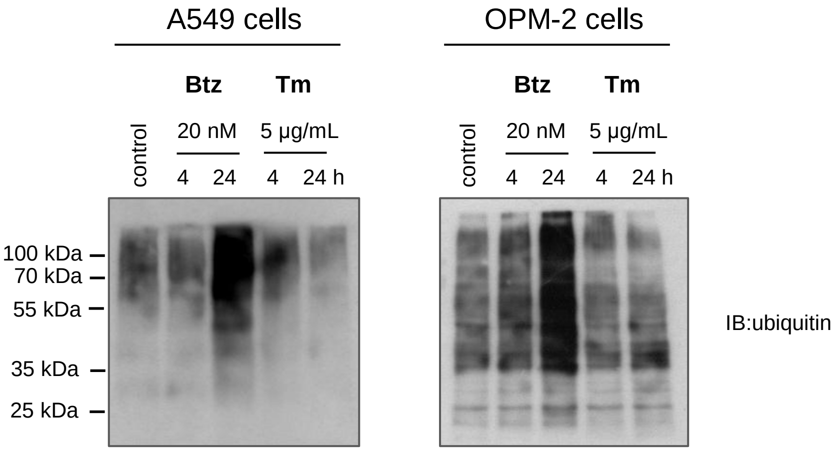

Supplementary Figure S3. Bortezomib disrupts the UPS in A549 cells. Representative immunoblots with antibodies against ubiquitin on whole cells extracts from A549 and OPM2 cells treated with bortezomib (Btz) or tunicamycin (Tm).

Supplementary Figure S4.

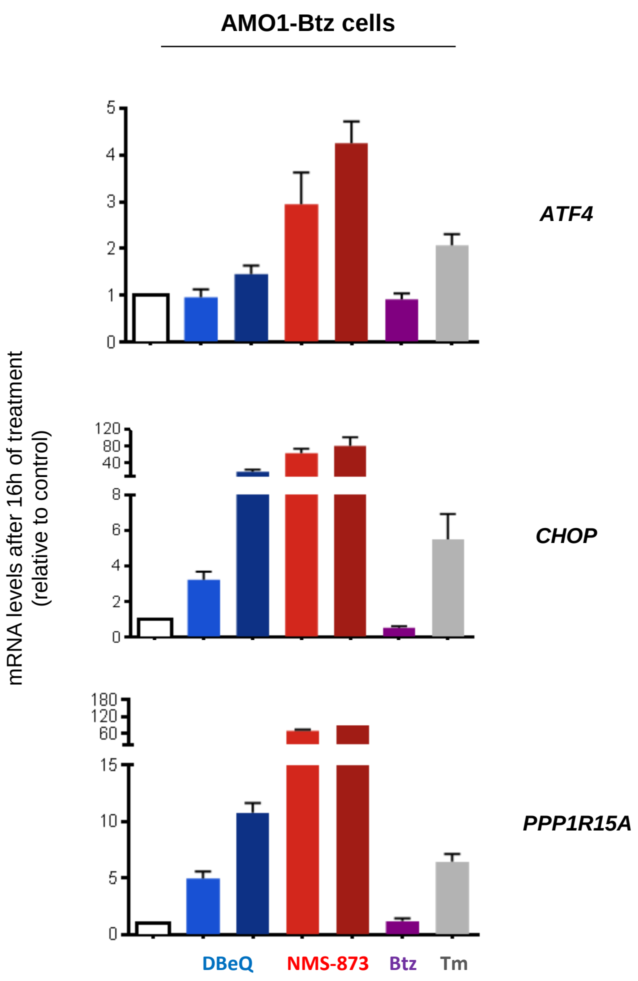

Supplementary Figure S4. VCP inhibition induces ATF4, CHOP, and PPP1R15A in bortezomib-adapted myeloma cells. mRNA levels of the indicated genes relative to untreated controls (white bars) determined by real-time quantitative PCR. Bortezomib-adapted AMO1-Btz cells were treated with DBeQ (10 and 15μM), NMS-873 (10 and 15μM), bortezomib (Btz, 20nM) or tunicamycin (Tm, 5μg/mL) for 16 h. Data shown are the mean ± SEM from 3 independent experiments.

Supplementary Figure S5.

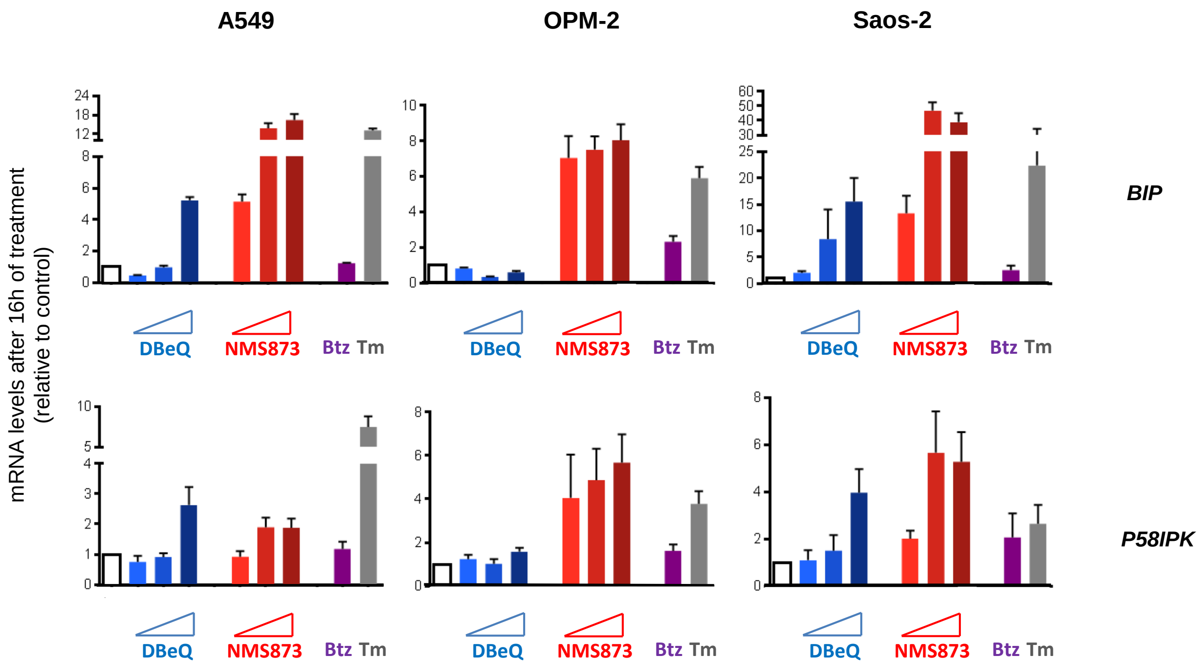

Supplementary Figure S5. VCP inhibitors induce ER chaperones. Relative mRNA levels of the indicated genes compared to untreated controls (white bars) determined by real-time quantitative PCR. The indicated cell lines were treated with DBeQ (5, 10, and 15μM), NMS-873 (5, 10, and 15μM), bortezomib (Btz, 20nM) or tunicamycin (Tm, 5μg/mL) for 16 h. Data shown are the mean ± SEM from 3 independent experiments.

Supplementary Figure S6.

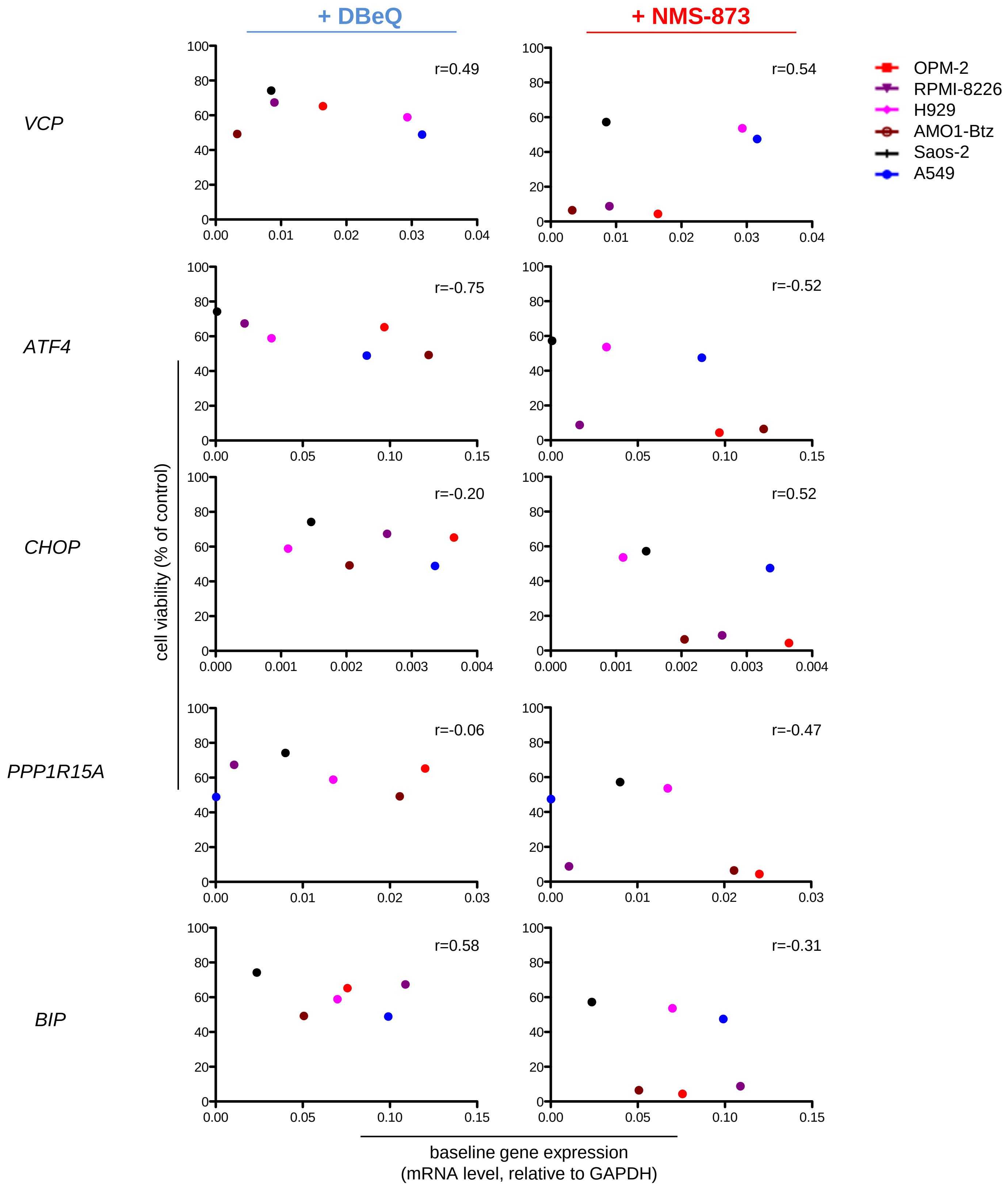

Supplementary Figure S6. VCP inhibitor-induced cell death does not correlate with the baseline expression of VCP or key proteotoxic stress response genes in cancer cell lines. Graphs show baseline mRNA levels of the indicated genes and correlation with cell death following treatment with DbeQ (10µM) or NMS-873 (10µM) for 24h. No r-value was statistically significant.

Supplementary Figure S7.

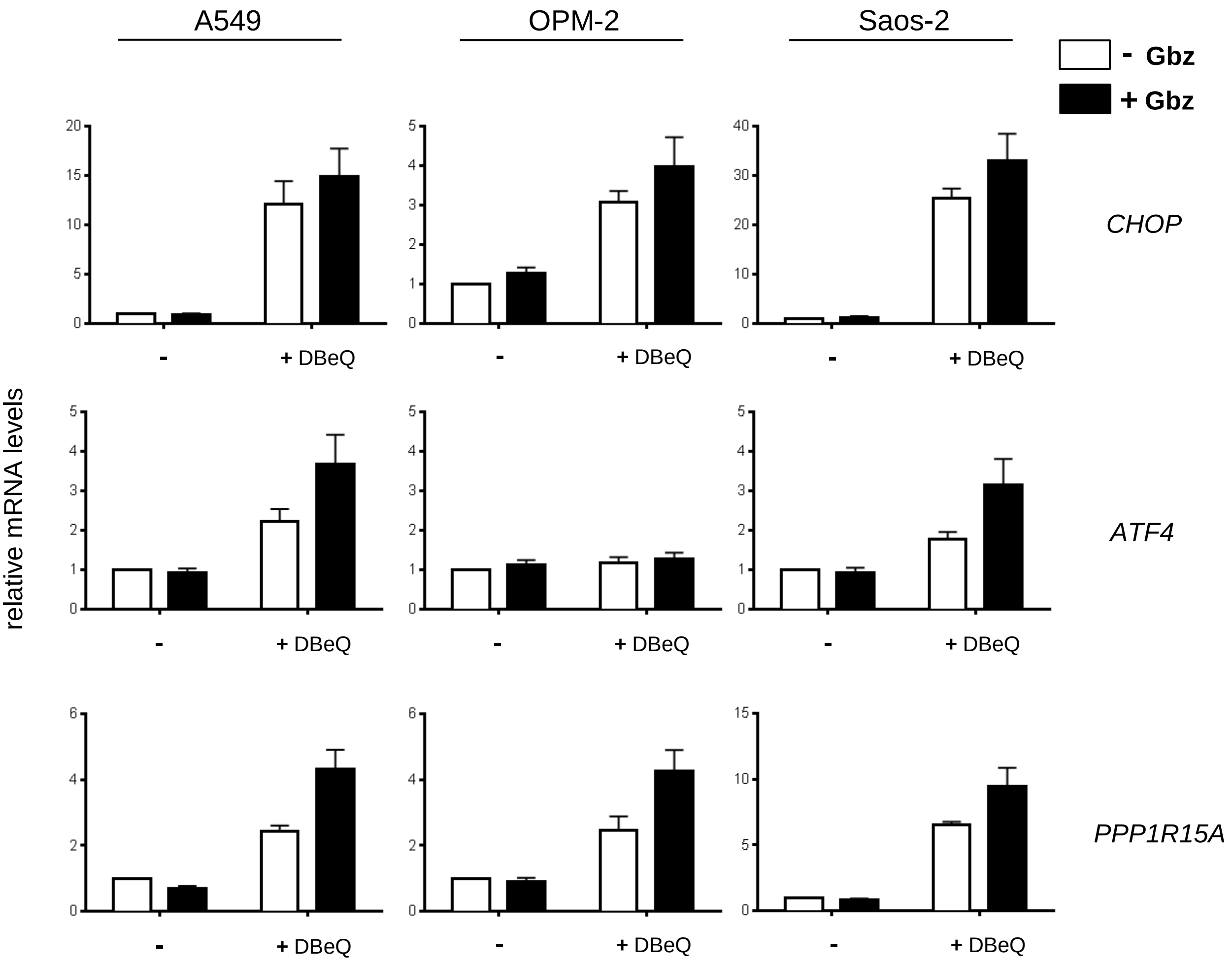

Supplementary Figure S7. Guanabenz impacts on mRNA levels of genes downstream of eIF2α. mRNA levels of the indicated genes relative to untreated controls determined by real-time quantitative PCR. A549, OPM-2, and Saos-2 cells were treated with DBeQ (15μM) in the presence of guanabenz (Gbz, 2.5μM) for 6h before mRNA isolation. Data shown are the mean ± SEM from 3 independent experiments.

Supplementary Figure S8.

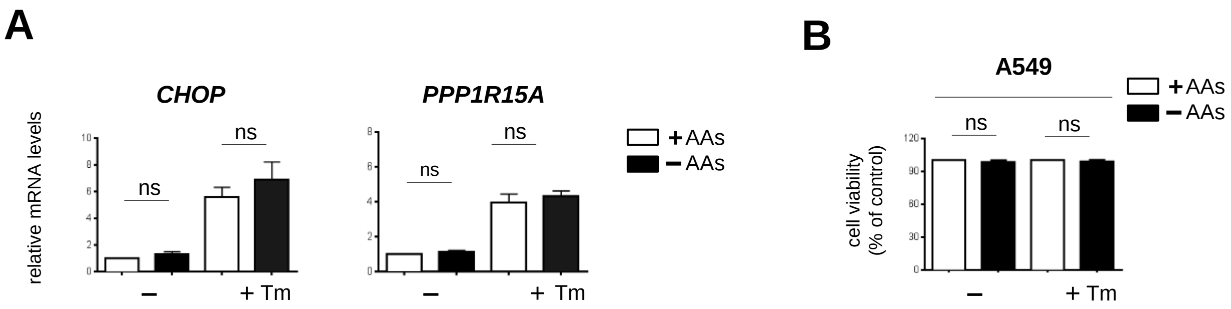

Supplementary Figure S8. Intracellular amino acid depletion has no effect on viability of A549 cells or induction of CHOP and PPP1R15A mRNAs after treatment with the glycosylation inhibitor tunicamycin. (A) Relative mRNA levels of the indicated genes in A549 cells grown in complete DMEM (+AA) or DMEM deficient in L-glutamine, L-methionine and L-cystine (-AA), and treated with tunicamycin (Tm, 5µg/mL) for 16h. **(B)** Viability of cells treated as in (A). Data shown are the mean ± SEM from 3 independent experiments; ns, not statistically significant.
