# Supplementary material for: Inadequate fine-tuning of protein synthesis and failure of amino acid homeostasis following inhibition of the ATPase VCP/p97
Source: Cell Death Dis. 2015 Dec 31;6(12):e2031–. doi: 10.1038/cddis.2015.373 (PMC4720905; doi:10.1038/cddis.2015.373)
Supplement: Supplementary Tables [file cddis2015373x2.ppt]

## Slide 1
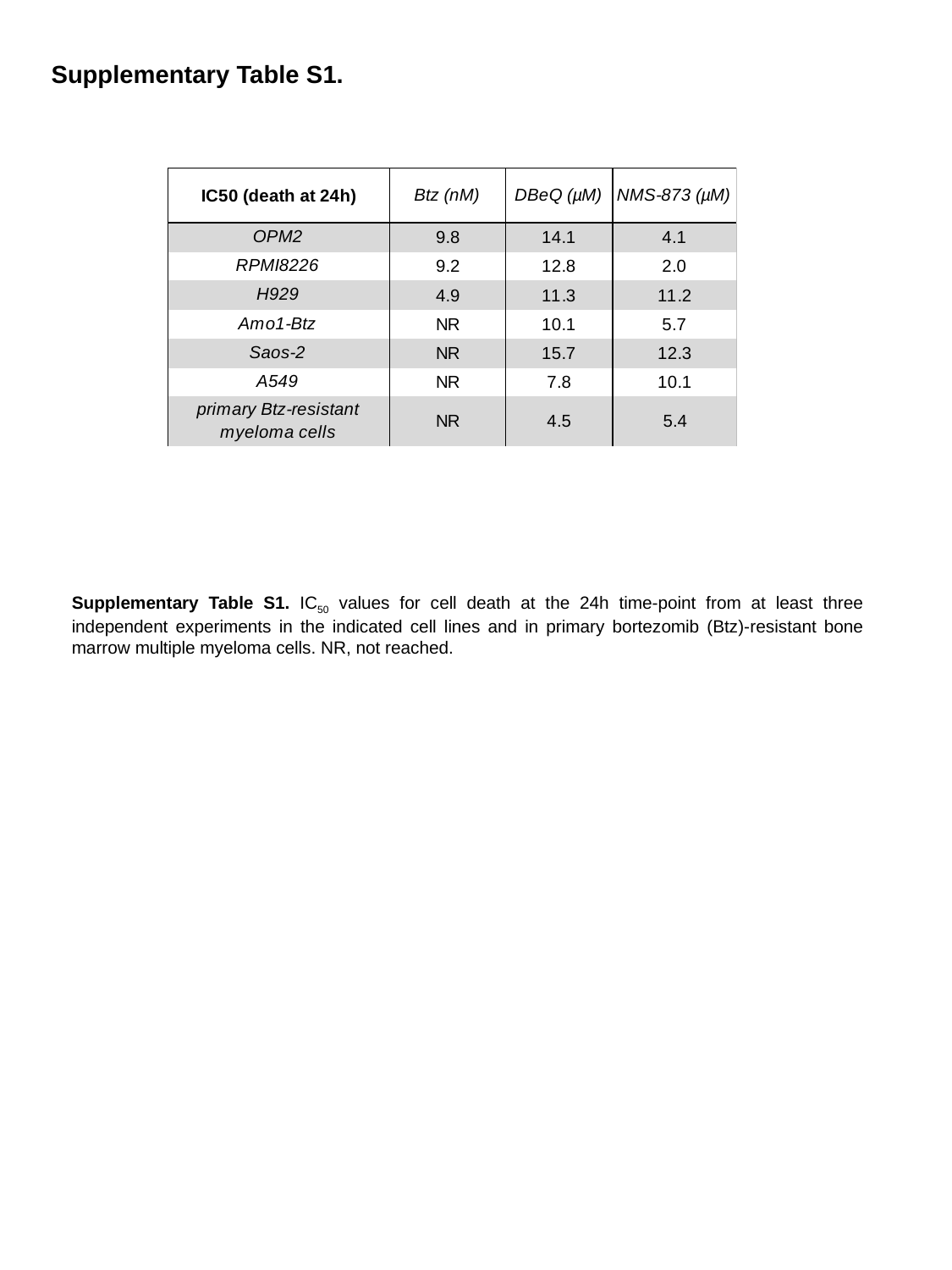

Supplementary Table S1.
Supplementary Table S1. IC50 values for cell death at the 24h time-point from at least three independent experiments in the indicated cell lines and in primary bortezomib (Btz)-resistant bone marrow multiple myeloma cells. NR, not reached.

## Slide 2
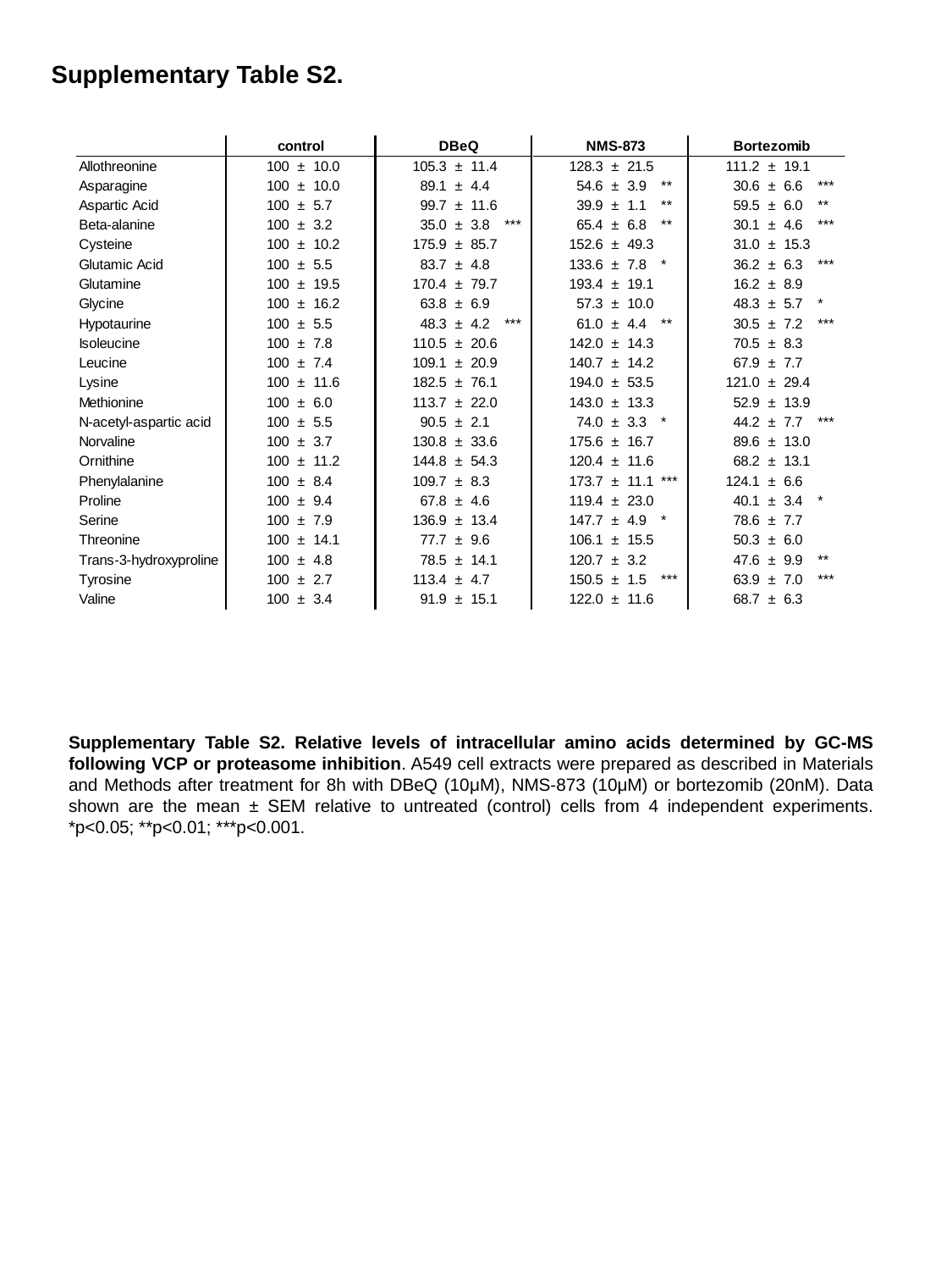

Supplementary Table S2.
Supplementary Table S2. Relative levels of intracellular amino acids determined by GC-MS following VCP or proteasome inhibition. A549 cell extracts were prepared as described in Materials and Methods after treatment for 8h with DBeQ (10μM), NMS-873 (10μM) or bortezomib (20nM). Data shown are the mean ± SEM relative to untreated (control) cells from 4 independent experiments. *p<0.05; **p<0.01; ***p<0.001.
